# Evolution of Aortic Valve Replacement Across Two Eras: Institutional Changes in Transcatheter and Surgical Practice and Outcomes

**DOI:** 10.3390/medsci14050558

**Published:** 2026-09-10

**Authors:** Jack T. Nickles, Marco Tagliafierro, Ezra Moos, Kenji Kaproth, Sarab Anand, Shirley Ge, Ali Fatehi Hassanabad, Luigi Pirelli

**Affiliations:** Division of Cardiothoracic Surgery, Columbia University Medical Center, 177 Fort Washington Avenue, 7GN-435, New York, NY 10032, USA; nicklesjack7@gmail.com (J.T.N.); fatehihassanabad@gmail.com (A.F.H.)

**Keywords:** transcatheter aortic valve replacement, surgical aortic valve replacement, aortic stenosis, case-mix migration, temporal trends, clinical outcomes, minimalist TAVR pathway

## Abstract

**Objectives:** Conventionally, the gold standard for aortic valve disease has been surgical aortic valve replacement (SAVR). Advances in transcatheter aortic valve replacement (TAVR) have since produced marked improvements in both outcomes and procedural volume. We sought to characterize this evolution by comparing the earliest and most recent 100 procedures of each modality at a single high-volume institution. **Methods:** We retrospectively compared four cohorts of 100 consecutive isolated procedures: 100 TAVRs from 2012 (the earliest period of complete and verifiable registry capture) and 100 first-time SAVRs from a contemporaneous period (2011), each versus the 100 most recent procedures of the same modality (TAVR 2024; SAVR 2024–2025). The SAVR cohorts were limited to first-time, isolated replacement; the TAVR cohorts to native-valve procedures. The primary endpoint was the 30-day composite of death or stroke. Secondary endpoints included new permanent pacemaker implantation, paravalvular leak, vascular access route, anesthetic technique, and length of stay. **Results:** For TAVR, the 30-day composite of death or stroke fell from 10% to 1% (*p* = 0.010), with fewer in-hospital deaths (6% to 0%; *p* = 0.029) and less new dialysis (4% to 0%; *p* = 0.059). A paravalvular leak of at least mild severity fell from 27% to 3% (*p* < 0.001), with none as moderate or greater in either era. New pacemaker implantation decreased from 15% to 6% (*p* = 0.056). Within the same comparison, general anesthesia decreased from 100% to 23%, non-transfemoral access from 29% to 2%, median ICU stay from 43 to 0 h, and hospital stay from 6 to 1 day (all *p* < 0.001). SAVR outcomes remained similar (composite 2% to 0%, *p* = 0.497; pacemaker 1% to 0%; and paravalvular leak 0% to 4%), while prolonged ventilation (11% to 1%; *p* = 0.007) and hospital stay (8 to 5 days; *p* < 0.001) improved. The SAVR population became younger (74 to 64 years; *p* < 0.001), and isolated first-time surgery was markedly less frequent, requiring 18.9 versus 5.7 months to accrue 100 consecutive cases. **Conclusions:** In this two-era institutional comparison, recent-era TAVR outcomes were markedly better, while SAVR outcomes remained excellent in both eras within a recent population that was younger and in whom eligible isolated first-time surgery was less frequent. Because the eras differed substantially in patient risk profile, these unadjusted within-modality comparisons describe a real-world redistribution of aortic stenosis care between two increasingly complementary treatments rather than isolating the effect of any single procedural or technological factor.

## 1. Introduction

Aortic stenosis (AS) remains the most common valvular heart disease in Western countries, affecting approximately 2% of adults over age 65 [1]. Surgical aortic valve replacement (SAVR) has long served as the definitive treatment and remains central to the management of patients with AS who can tolerate open cardiac surgery [2]. However, SAVR carries substantial operative risk in elderly patients and those with significant comorbidities, creating demand for a less invasive alternative for patients poorly suited to surgery.

Transcatheter aortic valve replacement (TAVR) has directly addressed this need. Since its FDA approval in 2011, TAVR has undergone iterative technological development and procedural maturation, expanding from a rescue therapy for inoperable and high-risk patients to a first-line therapy across the full range of risk profiles [3,4].

In this study, we describe 100 consecutive TAVR procedures performed at our institution during 2012, the earliest period of complete and verifiable registry capture at our institution following FDA approval of transcatheter aortic valve replacement in 2011, and compare baseline characteristics and outcomes to the most recent 100 TAVR cases at our institution. For comparison, 100 SAVR cases from the same early era are similarly compared to the 100 most recent SAVR cases, allowing us to characterize how patient selection and outcomes for both treatment modalities have evolved in parallel over this period.

National and multicenter data document a rapid temporal transformation of aortic valve replacement. Over the past decade, transcatheter procedures have risen sharply while surgical volumes for isolated procedures have fallen or remained relatively stable, such that TAVRs now account for the majority of aortic valve replacements performed in the United States [5]. Contemporary transcatheter populations have shifted toward lower surgical risk, and registry analyses show concurrent improvements in short-term mortality, length of stay, and procedural complications as devices, imaging, and periprocedural care have matured [6,7]. Current guidelines accordingly recommend a heart team, risk- and age-based approach to the choice between TAVR and SAVR across the spectrum of surgical risk [4]. Against this well-described national backdrop, detailed single-institution analyses that examine the simultaneous evolution of case selection, procedural workflow, and outcomes within both modalities remain relatively limited.

## 2. Methods

### 2.1. Patient Population and Data Collection

Following approval by the institutional IRB (IRB-ACYY0589), the data were collected retrospectively from the institutional Society of Thoracic Surgeons/American College of Cardiology Transcatheter Valve Therapy (STS/ACC TVT) database for transcatheter procedures, and from the New York State Cardiac Surgery Reporting System and the institutional STS Adult Cardiac Surgery Database for surgical procedures, each integrated with a retrospective chart review as appropriate. A total of 400 patients undergoing isolated aortic valve replacement at Columbia University Medical Center were included: the earliest 100 consecutively captured eligible patients undergoing isolated TAVRs in 2012, and the 100 most recent in 2024, together with 100 consecutive patients undergoing isolated first-time SAVRs over a contemporaneous early-era period (2011), and the 100 most recent in 2024–2025. Our institutional transcatheter program began in April 2011 under trial protocols, and systematic capture in the STS/ACC TVT registry began in April 2012. The early-era cohorts were, therefore, drawn from 2012 for TAVRs and 2011 for SAVRs, the earliest periods for which complete and verifiable data were available for each modality. Variables of interest were recorded through retrospective chart review, including patient demographics, past medical history, pre-operative clinical and echocardiographic characteristics, intraoperative details, readmission information, and mortality data. This study is reported in accordance with the STROBE (Strengthening the Reporting of Observational Studies in Epidemiology) guidance for observational research.

**Figure 1:**
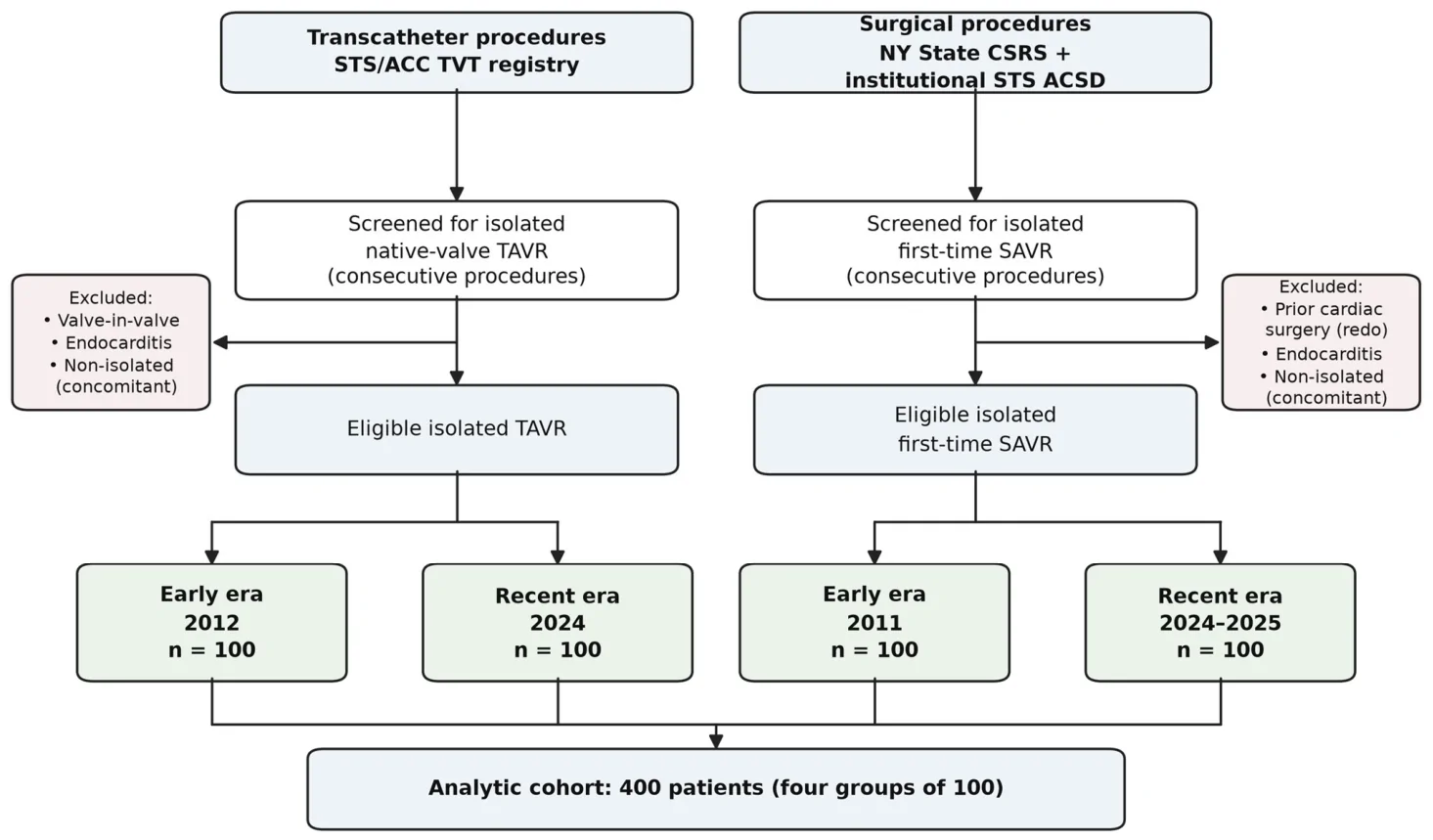
The patient flow diagram. Four cohorts of 100 consecutive isolated aortic valve replacements were derived from the STS/ACC TVT registry (TAVR), the New York State Cardiac Surgery Reporting System, and the institutional STS Adult Cardiac Surgery Database (SAVR), after exclusion of valve-in-valve, endocarditis, non-isolated, and, additionally for SAVR, prior cardiac surgery procedures.

Isolated aortic valve replacement was defined as aortic valve replacement without concomitant coronary artery bypass grafting, other valve repair or replacement, or aortic or other major cardiac procedures; concomitant surgical ablation for atrial fibrillation was permitted. Both SAVR cohorts were further restricted to first-time surgery, with no prior cardiac operation performed for non-infective aortic valve disease, thereby excluding active infective endocarditis. Both TAVR cohorts comprised isolated native-valve procedures, excluding valve-in-valve implantation and endocarditis. The early-era TAVR cohort comprised the earliest 100 consecutively captured eligible procedures in the STS/ACC TVT registry (April–November 2012); the recent-era TAVR cohort comprised the most recent 100 (September–December 2024). The early-era SAVR cohort comprised the first 100 consecutive eligible procedures of 2011 (January–July 2011), and the recent-era SAVR cohort comprised the most recent 100 (May 2024–December 2025). Patient selection in both eras was determined by a multidisciplinary heart team. The derivation of the four cohorts is summarized in Figure 1.

### 2.2. Primary and Secondary Endpoints

The primary endpoint was the 30-day composite of all-cause death or postoperative stroke. Secondary outcomes were new permanent pacemaker implantation and mild or greater PVL, together with in-hospital and 30-day mortality and postoperative stroke analyzed individually, new-onset renal failure requiring dialysis, prolonged mechanical ventilation (>24 h), reoperation for bleeding, ICU and hospital length of stay, 30-day readmission, vascular access route, and anesthetic technique.

The endpoints were defined to align with the Valve Academic Research Consortium-3 (VARC-3) framework [8] where the source data permitted. Death was all-cause and was reported both in-hospital (index admission) and at 30 days. Stroke was defined as a new focal or global neurological deficit consistent with a cerebrovascular event. Neurological events were recorded from the registries and confirmed by chart review, and were not independently adjudicated by a neurologist or imaging core laboratory. Paravalvular leak was assessed on the pre-discharge transthoracic echocardiogram, which was obtained and graded on the same standardized severity scale for all four cohorts and available for nearly all of the patients; all paravalvular comparisons are anchored at mild or greater. New dialysis denotes a new requirement for renal-replacement therapy after the procedure in a patient not receiving dialysis at baseline, and chronic-dialysis patients were excluded from this count. Reoperation for bleeding denotes a return to the operating room for bleeding or tamponade during the index admission for SAVRs, and unplanned surgical or endovascular reintervention for access-site or procedure-related bleeding for TAVRs. Readmission was any unplanned all-cause hospital admission within 30 days. Because registry versions and data sources differed between eras, the closest corresponding field was used for each variable and confirmed by chart review, and historical registry data precluded full VARC-3 harmonization for some endpoints. Thirty-day death, stroke, and readmission were ascertained through registry follow-up and institutional records supplemented by chart review; events occurring at other institutions after discharge may be incompletely captured.

### 2.3. Statistical Analysis

Individual analyses compared the earliest 100 and most recent 100 procedures within each modality. Continuous variables were summarized as the mean ± standard deviation (SD) when approximately normally distributed and as the median (IQR) otherwise, and were compared using the Student *t*-test or the Mann–Whitney U test, respectively. Between-era differences in continuous outcomes were estimated as Hodges–Lehmann median differences with 95% confidence intervals (CIs). Categorical variables were reported as counts and percentages, and compared using the Fisher exact test. Between-era differences in binary outcomes were expressed as absolute risk differences with Newcombe 95% confidence intervals. Analyses were performed using R version 4.5.1 (R Foundation for Statistical Computing, Vienna, Austria).

A propensity-score matching analysis was attempted to compare eras within each modality (1:1 nearest-neighbor matching, for age, sex, LVEF, creatinine, diabetes, chronic lung disease, peripheral vascular disease, prior cardiac surgery, baseline dialysis, and symptomatic status); however, adequate covariate balance and overlap could not be achieved, as approximately half of the patients lacked a suitable match. Therefore, matched results are not reported, and this study is presented as a descriptive, unadjusted comparison of institutional practice.

The study size was predetermined as a fixed sample of 100 consecutive procedures per era and per modality, chosen to preserve contemporaneous case-mix within each snapshot. The analysis is descriptive and hypothesis-generating. Estimates of infrequent events are potentially imprecise. Analyses used complete cases without imputation. Missing data were limited to a small number of variables concentrated in the earlier era, including the STS predicted risk of mortality (recorded for the contemporary cohorts only).

## 3. Results

### 3.1. Study Population and Design

This single-institution retrospective study comprised four cohorts of 100 isolated procedures each: early TAVRs (2012), recent TAVRs (2024), early SAVRs (2011), and recent SAVRs (2024–2025). Both SAVR cohorts were restricted to first-time, isolated surgical aortic valve replacement, excluding patients with prior cardiac surgery or active infective endocarditis. Both TAVR cohorts comprised isolated native-valve procedures, excluding valve-in-valve implantation and endocarditis. The interval required to accrue 100 consecutive procedures differed markedly between eras and modalities: 7.4 months for early TAVRs (April–November 2012) versus 3.2 months for recent TAVRs (September–December 2024), and 5.7 months for early SAVRs (January–July 2011) versus 18.9 months for recent SAVRs (May 2024–December 2025).

### 3.2. TAVR Baseline Characteristics

Patients undergoing TAVR in the early era (2012) were elderly and carried a heavy comorbidity burden (Table 1): the mean age was 82.2 ± 10.4 years and 48% were male, with chronic lung disease in 49%, peripheral vascular disease in 38%, and prior cardiac surgery in 34%. Baseline left ventricular ejection fraction (LVEF) was 51.4 ± 16.1%, and the median aortic valve peak gradient was 71 (60–82) mmHg. The STS predicted risk of mortality was not recorded during the early era, but all patients undergoing TAVR were explicitly labeled as “high- or prohibitive-risk” for surgery by a multidisciplinary heart team.

In the recent era of TAVRs, patients were more frequently male (64% vs. 48%, *p* = 0.033), with significantly less chronic lung disease (12% vs. 49%, *p* < 0.001) and peripheral vascular disease (9% vs. 38%, *p* < 0.001); higher LVEF (56.3 ± 13.4% vs. 51.4 ± 16.1%, *p* = 0.019); and lower aortic valve peak gradients (58 [44–74] vs. 71 [60–82] mmHg, *p* < 0.001). Recent-era patients had a median STS predicted risk of mortality of 5.1% [2.6–8.9]. Age (*p* = 0.129), diabetes (*p* = 0.305), and baseline dialysis (*p* = 1.000) did not differ significantly between eras.

All early-era TAVRs used the balloon-expandable Edwards SAPIEN valve (23–26 mm; Edwards Lifesciences, Irvine, CA, USA); recent-era TAVRs used the Sapien 3 Ultra RESILIA (n = 85; Edwards Lifesciences, Irvine, CA, USA), Medtronic Evolut (n = 11; Medtronic, Minneapolis, MN, USA), and Abbott Navitor (n = 3; Abbott, Santa Clara, CA, USA); and the device model was not available for the remaining patient.

### 3.3. SAVR Baseline Characteristics

Patients undergoing SAVRs in the early era (2011) were older (mean age 74.1 ± 11.0 years, 53% male); diabetes was present in 25%, chronic lung disease in 14%, and peripheral vascular disease in 10% (Table 1). In the recent era (2024–2025), patients undergoing SAVRs were markedly younger (64.4 ± 8.5 vs. 74.1 ± 11.0 years, *p* < 0.001), and had no peripheral vascular disease (0% vs. 10%, *p* = 0.004). Sex (57% vs. 53% male, *p* = 0.670), LVEF (*p* = 0.168), diabetes (*p* = 0.074), chronic lung disease (*p* = 0.446), and the aortic valve peak gradient (*p* = 0.085) were similar between eras. The STS predicted risk of mortality, available only for the recent era, was low, at 1.4% (0.9–1.9).

Surgical prostheses were bioprosthetic in all early-era SAVRs and in 95% of recent-era SAVRs, with five mechanical valves implanted in the recent era.

#### Procedural and Postoperative Outcomes

In the TAVR cohort, the primary composite of 30-day death or stroke decreased from 10% in the early era to 1% in the recent era (absolute difference −9.0%, 95% CI −16.5 to −2.7; *p* = 0.010; Table 2). Additional improvements included reductions in in-hospital mortality (6 vs. 0 deaths, *p* = 0.029) and 30-day mortality (7 vs. 1, *p* = 0.065), with the additional death in each era occurring after discharge. New dialysis, restricted to patients not receiving dialysis at baseline, trended lower (4/92 vs. 0/93 (4% vs. 0%), *p* = 0.059), as did postoperative stroke (3% vs. 0%, *p* = 0.246) and new PPM (15% vs. 6%, *p* = 0.056). PVL (mild or greater) decreased markedly, from 27% to 3% (*p* < 0.001), with no moderate or greater leak in either era. Unplanned intervention for post-operative bleeding was rare (2% vs. 0%, *p* = 0.497). Procedural practice changed markedly: access shifted from 71% transfemoral in the early era (with 26% transapical and 3% transaortic) to 98% transfemoral (*p* < 0.001), and anesthesia changed from 100% general sedation to 77% conscious or moderate sedation (*p* < 0.001). These practical changes resulted in the median ICU stay reducing from 43 (28–62) to 0 (0–0) hours (*p* < 0.001) and hospital length of stay from 6 (4–12) to 1 (1–2) days (*p* < 0.001). Thirty-day readmission was similar (4% vs. 6%, *p* = 0.749).

In the SAVR cohort, surgical outcomes were stable across eras: the composite of 30-day death or stroke (2% vs. 0%, *p* = 0.497), in-hospital mortality (1% vs. 0%), 30-day mortality (1% vs. 0%), stroke (1% vs. 0%), and new PPM rate (1% vs. 0%) all remained low. PVL (mild or greater) was 0% versus 4% (*p* = 0.121), with a single moderate leak in the recent era. Prolonged ventilation (11% vs. 1%, *p* = 0.007) and hospital length of stay (8 (6–14) vs. 5 (5–7) days, *p* < 0.001) improved, whereas reoperation for bleeding (8% vs. 2%, *p* = 0.105) did not differ significantly (Table 3).

## 4. Discussion

This is a retrospective evaluation of the earliest available 100 and the most recent 100 cases of both TAVR and SAVR at our institution, analyzed within each modality and compared across an early era (TAVR 2012; SAVR 2011) and a contemporary era (TAVR 2024; SAVR 2024–2025). In brief, recent-era TAVR outcomes were markedly better as the 30-day composite of death or stroke fell from 10% to 1% (*p* = 0.010), with lower in-hospital mortality, less paravalvular leak, and a shift toward transfemoral, sedation-based, ICU-free care, whereas SAVR outcomes remained excellent and largely unchanged, even as perioperative recovery improved and the surgical population became younger and eligible isolated first-time surgery less frequent. National and multicenter analyses have already documented the broader temporal transition in TAVR and SAVR utilization and outcomes [5,6,7]. This analysis is narrower and complementary: a deliberately symmetric, single-institution bookend comparison that shows how this national transition manifested inside one mature, high-volume program at the patient, procedural, outcome, and workflow levels simultaneously.

The observed improvement in institutional TAVR outcomes likely reflects the combined effects of markedly altered patient selection, device evolution, procedural maturation, changes in access and anesthesia strategy, and contemporary periprocedural care. The present design cannot weigh the contribution of any single factor. Randomized trials established transcatheter non-inferiority to surgery across high-, intermediate-, and low-risk populations [9,10,11,12,13,14], and transcatheter volume subsequently exceeded surgical volume nationally [15]. The earliest iteration of TAVR was performed on an older and sicker population who were surgically prohibitive [16]. Consistent with the indications of the time, all early-era patients were deemed high-risk or inoperable for surgery by a multidisciplinary heart team. Over the study period, the multidisciplinary selection process itself was maintained, but eligibility broadened in line with accumulating trial evidence, evolving guidelines, and expanding regulatory indications, from high- or prohibitive-risk patients in the early era to patients across the full spectrum of surgical risk in the recent era, itself a principal driver of the observed case-mix shift. A clear example of this evolution is our access data: in the early era only 71 of 100 TAVRs were transfemoral (26 transapical and 3 transaortic), whereas 98 of 100 were transfemoral in the recent era (*p* < 0.001), reflecting a progressive shift away from high-morbidity alternative-access routes. Broader refinements in imaging, planning, and technique have been reported to improve the safety of TAVR and may have contributed here, although our study was not designed to attribute outcomes to any individual practice change. Because the early and contemporary procedures were not necessarily performed by the same operators, these gains are best understood as maturation of the institutional TAVR program, which encompasses the heart team, imaging, patient selection, and workflow, rather than an individual operator learning curve.

Beyond these procedural refinements, the most striking institutional change was in the periprocedural care pathway itself. Four independent measures moved in concert: general anesthesia gave way to conscious or moderate sedation in 77% of recent cases, alternative access fell from 29% to 2%, median intensive care unit stay decreased from 43 h to 0 h, and median hospital stay fell from 6 days to 1 day (all *p* < 0.001). Indeed, 87 of 99 recent-era patients had no ICU stay whatsoever, proceeding directly from the catheterization laboratory to a recovery area and then a telemetry floor before discharge, with intensive care reserved for the 12 of 99 who required overnight monitoring for arrhythmia or transient instability. This constellation reflects the maturation of the minimalist TAVR pathway [17], in which a transfemoral, sedation-based procedure in an anatomically screened patient permits recovery outside the intensive care setting. Critically, these changes occurred alongside, rather than at the expense of, the observed reductions in mortality and renal complications.

Surgery, by contrast, entered the study period as an already mature operation whose technical conduct was highly optimized, leaving little room for the stepwise gains seen with TAVR, and much of its subsequent innovation has occurred in prosthesis design rather than in the fundamental operative approach. Accordingly, surgical mortality was already low and remained unchanged (1% to 0%), with stable rates of postoperative stroke, pacemaker implantation, and reoperation for bleeding. Between eras, perioperative recovery improved (prolonged ventilation 11% to 1%, *p* = 0.007; length of stay 8 to 5 days, *p* < 0.001), consistent with general advances in perioperative care rather than a change in the operation itself. The more informative surgical signals were, therefore, in case selection and volume. The two modalities evolved toward different patient profiles: TAVR expanded into progressively lower-risk populations, whereas isolated SAVR became less frequent and was increasingly performed in younger patients with a low predicted risk of mortality (recent median for the STS predicted risk of mortality of 1.4%). This redistribution was also evident in accrual, as the 100 consecutive isolated first-time SAVRs required 5.7 months in 2011 but 18.9 months in 2024–2025, whereas 100 consecutive isolated TAVRs accrued in 3.2 months in the recent era compared with 7.4 months in 2012. Annual institutional volumes across the study period confirm this redistribution, with isolated first-time SAVRs falling from approximately 40% to 12% of all aortic valve replacements while the total volume remained broadly stable (Supplementary Figure S1). Whether the surgical cases that remain also represent a more complex anatomic substrate, such as bicuspid disease, aortopathy, or concomitant pathology, is a plausible but untestable hypothesis in the present design, as concomitant procedures were excluded and valve morphology was not systematically captured.

Taken together, these trajectories position TAVR and SAVR as complementary rather than directly competing therapies across the spectrum of aortic stenosis. As short-term outcomes for both have become excellent, the decisive questions have shifted from immediate procedural safety to lifetime valve management, particularly as transcatheter therapy extends to younger patients. Surgical bioprostheses have demonstrated durability beyond two decades [18,19], and mid-term transcatheter results are encouraging [20,21], but a lifetime perspective must also weigh the consequences of the first valve choice on subsequent options. These include the feasibility of redo transcatheter intervention (TAVR-in-TAVR) and of surgical explant after a failed transcatheter valve, the preservation of coronary access for future percutaneous intervention, an increasingly important consideration given contemporary device designs and commissural-alignment strategies, prosthesis–patient mismatch, the cumulative burden of conduction disturbance and permanent pacemaker implantation across sequential procedures, and the management of bicuspid anatomy in younger candidates. Prospective, longer-term comparative data incorporating these lifetime-management endpoints will be needed to define the optimal sequencing of transcatheter and surgical therapy for individual patients.

### Limitations

This study has several limitations. First, it is a single-center, retrospective analysis of two fixed 100-patient samples and is descriptive and exploratory; as such, its findings may not generalize to lower-volume institutions. Second, differences in early and contemporary cohorts cannot be attributed to any single cause: changes in patient selection, device iteration, imaging and procedural technique, access and anesthesia strategy, periprocedural care, and case mix are summative and cannot be isolated in this design. Standardized baseline risk (STS predicted risk of mortality) was not available for the historical cohorts, so comparable risk adjustment across eras is not possible, and the outcome differences cannot be cleanly separated from changes in patient selection. A propensity-score matching analysis was attempted (Section 2.3) but is not reported because adequate covariate balance and overlap could not be achieved; the inability to achieve adequate propensity-score overlap further illustrates the substantial differences in case mix between eras.

Several specific limitations also apply. Because the early cohorts carried substantially higher mortality, death may act as a competing event for stroke within the 30-day window. Although we report the timing of deaths (in-hospital versus post-discharge), cause of death was not reliably available, and thus not analyzed. Our volume comparison reflects the slower accrual of eligible isolated first-time SAVRs rather than a denominator-based measure of overall surgical activity; contemporary aortic valve surgery increasingly occurs with concomitant coronary, aortic or root, mitral, redo, or endocarditis procedures that were excluded by design, and complete annual institutional denominators for all aortic valve replacement were not analyzed here. Because only 100 patients were studied per era, rare events may have been missed. Valve morphology was not systematically available for the early cohorts; where recorded, bicuspid disease accounted for 17% of contemporary SAVRs, consistent with a younger surgical population, and a systematic morphologic comparison across eras was not possible. Finally, follow-up was limited to in-hospital and 30-day endpoints, precluding assessment of durability, reintervention, or long-term survival, and events occurring at other institutions after discharge may be incompletely captured.

## 5. Conclusions

In this two-era institutional comparison, recent-era TAVR outcomes were markedly better than its earliest iterations, with lower rates of the 30-day composite of death or stroke, in-hospital mortality, paravalvular leak, new dialysis, shorter length of stay, and a lower rate of new pacemaker implantation, accompanied by a transformation of the periprocedural pathway toward transfemoral, sedation-based, intensive-care-free care, in a progressively lower-risk population. SAVR outcomes remained excellent in both eras, while the surgical population became younger and eligible isolated first-time SAVRs became less frequent. These divergent trajectories are consistent with the maturation of transcatheter therapy and the established reliability of surgery, but they also reflect a substantial redistribution of the aortic stenosis population and a marked shift in case mix between eras. As TAVR indications continue to broaden to younger and lower-risk patients, longer-term comparative data on durability and reintervention will be essential to define the appropriate role of each therapy across the full lifetime management spectrum.

## Figures and Tables

**Table 1:** Baseline characteristics.

	TAVR			SAVR		
	Early (n = 100)	Recent (n = 100)	*p* Value	Early (n = 100)	Recent (n = 100)	*p* Value
Age, years	82.2 ± 10.4	80.1 ± 8.2	0.129	74.1 ± 11.0	64.4 ± 8.5	<0.001
Male sex, n (%)	48 (48)	64 (64)	0.033	53 (53)	57 (57)	0.670
LVEF, %	51.4 ± 16.1	56.3 ± 13.4	0.019	57.1 ± 9.6	59.0 ± 10.1	0.168
Creatinine, mg/dL	1.1 (0.9–1.6)	1.1 (0.9–1.4)	0.678	0.9 (0.8–1.2)	0.9 (0.7–1.1)	0.122
Diabetes mellitus, n (%)	33 (33)	41 (41)	0.305	25 (25)	14 (14)	0.074
Chronic lung disease, n (%)	49 (49)	12 (12)	<0.001	14 (14)	19 (19)	0.446
Peripheral vascular disease, n (%)	38 (38)	9 (9)	<0.001	10 (10)	0 (0)	0.004
Prior cardiac surgery, n (%)	34 (34)	21 (21)	0.057	0 (0)	0 (0)	–
Baseline dialysis, n (%)	8 (8)	7 (7)	1.000	1 (1)	0 (0)	1.000
Symptomatic, n (%)	98 (98)	95/99 (96)	0.445	84 (84)	66/90 (73)	0.105
Aortic valve peak gradient, mmHg	71 (60–82)	58 (44–74)	<0.001	77 (57–83)	66 (56–80)	0.085
STS predicted risk of mortality, %	NR	5.1 (2.6–8.9)	–	NR	1.4 (0.9–1.9)	–

The data are the mean ± SD, median [IQR], or n (%); n/N (%) is shown where the denominator differs from 100. *p* values compare the early versus recent era within each modality (*t*-test, Mann–Whitney U, or Fisher’s exact test). LVEF, left ventricular ejection fraction; NR, not recorded; STS, Society of Thoracic Surgeons.

**Table 2:** Procedural characteristics and outcomes.

	TAVR				SAVR			
	Early (n = 100)	Recent (n = 100)	Difference (95% CI)	*p* Value	Early (n = 100)	Recent (n = 100)	Difference (95% CI)	*p* Value
Composite: 30-day death or stroke, n (%)	10 (10)	1 (1)	−9.0 (−16.5 to −2.7)	0.010	2 (2)	0 (0)	−2.0 (−7.0 to 2.0)	0.497
In-hospital death, n (%)	6 (6)	0 (0)	−6.0 (−12.5 to −1.1)	0.029	1 (1)	0 (0)	−1.0 (−5.4 to 2.8)	1.000
30-day death, n (%)	7 (7)	1 (1)	−6.0 (−12.8 to −0.3)	0.065	1 (1)	0 (0)	−1.0 (−5.4 to 2.8)	1.000
Postoperative stroke, n (%)	3 (3)	0 (0)	−3.0 (−8.5 to 1.2)	0.246	1 (1)	0 (0)	−1.0 (−5.4 to 2.8)	1.000
New permanent pacemaker, n (%)	13/86 (15)	6/96 (6)	−8.9 (−18.5 to 0.2)	0.056	1/98 (1)	0/99 (0)	−1.0 (−5.6 to 2.8)	0.497
Paravalvular leak/AI ≥ mild, n (%)	25/94 (27)	3/88 (3)	−23.2 (−33.2 to −13.2)	<0.001	0 (0)	4 (4)	4.0 (−0.4 to 9.8)	0.121
Paravalvular leak/AI ≥ moderate, n (%)	0/94 (0)	0/88 (0)	0.0 (−3.9 to 4.2)	1.000	0 (0)	1 (1)	1.0 (−2.8 to 5.4)	1.000
New dialysis, n (%)	4/92 (4)	0/93 (0)	−4.3 (−10.7 to 0.4)	0.059	1/99 (1)	0 (0)	−1.0 (−5.5 to 2.8)	0.497
Reoperation for bleeding, n (%)	2 (2)	0 (0)	−2.0 (−7.0 to 2.0)	0.497	8 (8)	2 (2)	−6.0 (−13.1 to 0.3)	0.101
Prolonged ventilation > 24 h, n (%)	–	–	–	–	11 (11)	1 (1)	−10.0 (−17.7 to −3.5)	0.005
30-day readmission, n (%)	4/93 (4)	6 (6)	1.7 (−5.3 to 8.7)	0.749	5 (5)	1 (1)	−4.0 (−10.2 to 1.3)	0.212
Vascular access route				<0.001	–	–	–	–
Transfemoral	71 (71)	98 (98)			–	–	–	–
Transapical	26 (26)	0 (0)			–	–	–	–
Transaortic	3 (3)	0 (0)			–	–	–	–
Trans-axillary/subclavian	0 (0)	2 (2)			–	–	–	–
General anesthesia, n (%)	100 (100)	23 (23)	−77.0 (−84.2 to −67.1)	<0.001	100 (100)	100 (100)	0.0 (−3.7 to 3.7)	1.000
Conscious/moderate sedation, n (%)	0 (0)	77 (77)	77.0 (67.1 to 84.2)	<0.001	–	–	–	–
ICU stay, hours	43 (28–62)	0 (0–0)	−35.5 (−47.7 to −31.7)	<0.001	48 (24–96)	40 (26–70)	−5.9 (−35.4 to 2.0)	0.050
Hospital length of stay, days	6 (4–12)	1 (1–2)	−5.0 (−7.0 to −4.0)	<0.001	8 (6–14)	5 (5–7)	−2.0 (−4.5 to −1.0)	<0.001

The data are n (%) and n/N (%), where the denominator differs from 100, or the median [IQR]. Differences are absolute risk differences, each calculated as recent minus early. Prolonged ventilation applies to surgical (SAVR) procedures; conscious/moderate sedation applies to TAVRs. AI, aortic insufficiency; CI, confidence interval; ICU, intensive care unit.

**Table 3:** A summary of the divergent evolution of transcatheter (TAVR) and surgical (SAVR) aortic valve replacement across the early and contemporary eras.

	TAVR		SAVR	
	Early Era	Contemporary Era	Early Era	Contemporary Era
Patient population	Inoperable/high-risk, elderly	Lower-risk, broader eligibility	Standard-risk candidates	Younger, low-risk
Predicted risk (STS-PROM)	NR	~5%	NR	~1.4%
Device/prosthesis	First-generation balloon-expandable	Balloon-expandable + self-expanding	Bioprosthetic (tissue)	Bioprosthetic (95%)
Access/approach	71% transfemoral	98% transfemoral	Open (sternotomy)	Open (sternotomy)
Anesthesia	General (100%)	Sedation (77%)	General	General
Recovery/ICU	Routine ICU (~43 h)	ICU-free (~0 h)	Routine ICU	Routine ICU
Length of stay	6 days	1 day	8 days	5 days
Accrual of 100 cases	7.4 months	3.2 months	5.7 months	18.9 months

## Data Availability

The data presented in this study are not publicly available because they contain protected health information and are governed by institutional and registry (STS/ACC TVT and New York State Cardiac Surgery Reporting System) data-use agreements. De-identified data may be available from the corresponding author upon reasonable request and with the permission of the relevant institutional and registry authorities.

## Supplementary Materials

The following supporting information can be downloaded at https://www.mdpi.com/article/10.3390/medsci14050558/s1, Figure S1, Annual Aortic Valve Replacement Volume at a High Volume Center.
