# Supplementary material for: Evolution of Aortic Valve Replacement Across Two Eras: Institutional Changes in Transcatheter and Surgical Practice and Outcomes
Source: Med Sci (Basel). 2026 Sep 10;14(5):558. doi: 10.3390/medsci14050558 (PMC13609362; doi:10.3390/medsci14050558)
Supplement: Supplementary file 1 [file medsci-14-00558-s001.zip › medsci-4511003-supplementary.pdf]

medical sciences

MDPI

Article

Evolution of Aortic Valve Replacement Across Two Eras: Institutional Changes in TAVR and SAVR Practice and Outcomes

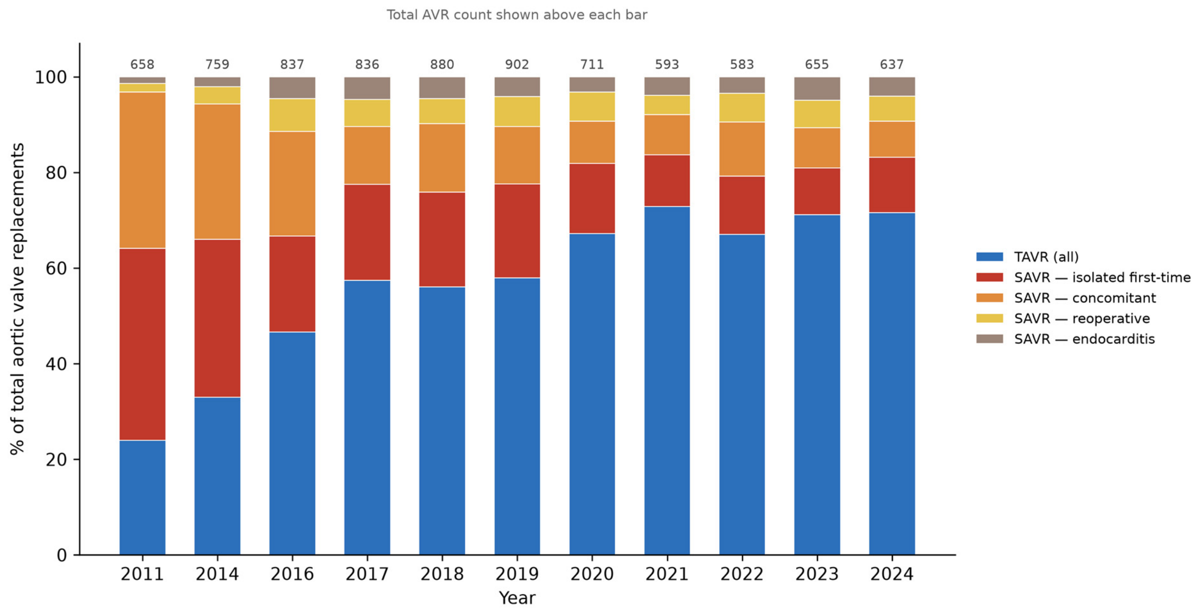

Figure S1. Annual Aortic Valve Replacement Volume at a High Volume Center.
